# Comparative Analysis of Neuropsychiatric Adverse Reactions Associated with Remdesivir and Nirmatrelvir/Ritonavir in COVID-19 Treatment: Insights from EudraVigilance Data

**DOI:** 10.3390/jcm14061886

**Published:** 2025-03-11

**Authors:** Aliteia-Maria Pacnejer, Mihaela Cristina Negru, Anca Maria Arseniu, Cristina Trandafirescu, Cristian Oancea, Felicia Gabriela Gligor, Claudiu Morgovan, Anca Butuca, Cristina Adriana Dehelean

**Affiliations:** 1 Department of Toxicology, Drug Industry, Management and Legislation, Faculty of Pharmacy, “Victor Babeș” University of Medicine and Pharmacy, 2nd Eftimie Murgu Square, 300041 Timișoara, Romania; aliteia.pacnejer@umft.ro (A.-M.P.); cadehelean@umft.ro (C.A.D.); 2 Preclinical Department, Faculty of Medicine, “Lucian Blaga” University of Sibiu, 550169 Sibiu, Romania; felicia.gligor@ulbsibiu.ro (F.G.G.); claudiu.morgovan@ulbsibiu.ro (C.M.); anca.butuca@ulbsibiu.ro (A.B.); 3 Department of ENT, “Victor Babeș” University of Medicine and Pharmacy, Eftimie Murgu Square No. 2, 300041 Timișoara, Romania; 4 Discipline of Pharmaceutical Chemistry, Faculty of Pharmacy, “Victor Babeș” University of Medicine and Pharmacy, 2nd Eftimie Murgu Square, 300041 Timișoara, Romania; trandafirescu.cristina@umft.ro; 5 Department of Pulmonology, Center for Research and Innovation in Personalized Medicine of Respiratory Diseases, “Victor Babeș” University of Medicine and Pharmacy, 300041 Timișoara, Romania; oancea@umft.ro; 6 Research Center for Pharmaco-Toxicological Evaluations, Faculty of Pharmacy, “Victor Babeș” University of Medicine and Pharmacy, Eftimie Murgu Square No. 2, 300041 Timișoara, Romania

**Keywords:** COVID-19, remdesivir, nirmatrelvir/ritonavir, neuropsychiatric adverse effects, pharmacovigilance, EudraVigilance

## Abstract

Remdesivir (RDV) and nirmatrelvir/ritonavir (NMVr) are among the most widely used antivirals in the treatment of COVID-19, aiming to reduce disease severity and progression. Adverse neuropsychiatric effects, such as anxiety, sleep disturbances, and movement disorders, have emerged as significant concerns associated with these treatments. To better understand the safety profiles of RDV and NMVr, this study performs a pharmacovigilance analysis of individual case safety reports (ICSRs) from the EudraVigilance (EV) database. **Objectives:** This study evaluates the risk of neuropsychiatric adverse events associated with RDV and NMVr. Comparisons with other antiviral drugs, including darunavir, sofosbuvir, ribavirin, tenofovir, ritonavir, and sotrovimab, are also performed to develop a comprehensive understanding of the safety profiles. **Methods:** A retrospective analysis of ICSRs submitted to EV until 7 July 2024, with data extraction on 12 July 2024, was conducted. Demographic characteristics (age, sex, geographic region, and reporter type) and case severity were included in the descriptive analysis. Disproportionality analysis using reporting odds ratio (ROR) and 95% confidence intervals (CI) was performed to compare adverse drug reaction (ADRs) frequencies across 27 system organ classes (SOCs), with emphasis on “Nervous system disorders” and “Psychiatric disorders. **Results:** The total number of ICSRs was significantly higher for NMVr (*n* = 8078) compared to RDV (*n* = 3934). Nervous system disorders accounted for 3.07% of the total RDV reports and for 17.31% of NMVr reports, while psychiatric disorders represented 0.92% of the total ADRs reported for RDV (*n* = 60) and 3.61% for NMVr (*n* = 672). On the other hand, RDV showed a significantly lower frequency of reporting headache compared to NMVr (ROR: 0.1057; 95% CI: 0.0676–0.1653). **Conclusions:** NMVr presents a higher risk of neuropsychiatric ADRs than RDV, underscoring the need for enhanced monitoring, particularly in patients with preexisting central nervous system (CNS) conditions. These findings contribute to optimizing antiviral safety and informing clinical decision making.

## 1. Introduction

The COVID-19 pandemic caused by severe acute respiratory syndrome coronavirus 2 (SARS-CoV-2) significantly affected global health and healthcare systems, resulting in an unprecedented burden on medical resources. The crisis underscored the urgent need for rapid development and delivery of effective therapies to reduce disease severity and mortality. In addition to its respiratory manifestations, the infection with the SARS-CoV-2 virus has been associated with significant neurological and psychiatric complications, including anxiety, depression, delirium, and cognitive impairment. These effects are associated with direct viral invasion of the CNS, systemic inflammation, hypoxia, and hypercoagulability [1,2,3].

The neuropsychiatric manifestations of COVID-19 further complicate an already complex landscape, as the virus itself has been shown to increase the risk of anxiety, depression, and other CNS-related effects. Studies suggest that up to 36% of hospitalized COVID-19 patients experience neurological or psychiatric symptoms, making it difficult to distinguish between disease-related and drug-induced effects [1,4]. The neuroinvasive capacity of SARS-CoV-2, driven by mechanisms such as cytokine-induced neuroinflammation, endothelial damage, and direct viral entry into neural tissue, has been extensively documented. These pathways contribute to a spectrum of neuropsychiatric manifestations, including mood disturbances, cognitive impairments, and persistent fatigue, further highlighting the need for comprehensive evaluation and monitoring of CNS outcomes in COVID-19 patients [5].

Antiviral treatments have played a central role in combating the pandemic, particularly in reducing disease severity and progression to critical illness. Among these, RDV and NMVr have been widely used in clinical practice.

RDV, a nucleotide analogue that inhibits RNA-dependent RNA polymerase (RdRp), is essential for inhibiting viral replication. It was the first antiviral approved for emergency use during the pandemic, efficient in reducing hospitalizations and improving outcomes in patients with severe COVID-19 [6,7,8,9,10]. NMVr is a combination of a 3CL protease inhibitor, nirmatrelvir, and a CYP3A4 inhibitor, ritonavir, which improves the pharmacokinetics of nirmatrelvir. This treatment is indicated for high-risk, non-hospitalized patients and has shown significant efficacy in reducing hospitalizations and deaths when initiated early in the course of the disease [11]. Despite their clinical benefits, the safety profiles of these treatments, particularly regarding neuropsychiatric adverse events, remain a critical area of investigation.

Both RDV and NMVr are associated with ADR, although the nature and frequency of these reactions vary. RDV is primarily associated with systemic adverse effects, including hepatotoxicity, renal dysfunction, and cardiac events, but occasional reports of delirium and encephalopathy have raised concerns about its effects on the CNS [12,13,14,15]. NMVr, due to the ability of ritonavir to inhibit CYP3A4, has an increased risk of drug–drug interactions leading to neuropsychiatric adverse events such as anxiety, insomnia, and cognitive dysfunction [16,17].

Despite the available evidence, significant gaps remain in the understanding of neuropsychiatric adverse effects associated with these antiviral drugs. Current research has focused primarily on systemic adverse effects, with limited comparative analysis of the neuropsychiatric effects of RDV and NMVr. Evidence from other antiviral agents have shown neuropsychiatric ADRs, providing a context for understanding these effects in COVID-19 treatments. For example, efavirenz, used in the treatment of HIV, is known to have psychotropic effects, including vivid dreams, hallucinations, and mood disturbances. These effects are related to efavirenz’s interaction with serotonin receptors and monoaminergic neurotransmission [18]. Protease inhibitors such as lopinavir–ritonavir and darunavir have shown neurotoxic effects, including peripheral neuropathy and depressive symptoms [19,20,21]. Ritonavir-boosted darunavir has been associated with neuropsychiatric events such as headache and depression [17]. The main objective of this study is to identify and assess the risk of neuropsychiatric ADRs associated with the administration of RDV and NMVr. Additionally, it aims to provide a descriptive analysis of the ICSR data regarding these reactions, focusing on remdesivir and NMVr, including a disproportionality analysis of adverse reactions.

## 2. Materials and Methods

### 2.1. Data Analysis

This study evaluates the safety profiles of two drugs used in the treatment of COVID-19: RDV and the combination NMVr. ICSRs from the EV database, uploaded until 7 July 2024, were included in the analysis. Data extraction was conducted on 12 July 2024 from the EV platform (https://www.adrreports.eu/en/search_subst.html). Web reports on the side effects of the selected drugs in the study were searched in the “suspected adverse drug reaction reports for substances” tab using the A–Z browse tool. All reports follow the same format, contain the same functions, and include the same data fields. The data used in our study were extracted from the following sections: “Number of individual cases”, “Number of individual cases by reaction groups” (where data were selected based on reaction group, seriousness, reporter group, and geographic origin), and “Number of individual cases for a selected reaction”, where individual cases for a user-defined selected reaction were collected.

### 2.2. Descriptive Analysis

The descriptive analysis focused on the ICSRs submitted for RDV and NMVr in the EV database. It included demographic information such as patient age (categorized into eight groups: not specified (NS), 0–1 month, 2 months–2 years, 3–11 years, 12–17 years, 18–64 years, 65–85 years, and over 85 years) and sex (female, male, and NS). The characteristics of the reporters, including professional background (healthcare professionals (HPs), non-HPs, or NS) and geographical region (European Economic Area (EEA), non-EEA, or NS), were also analyzed. Considering that a single ICSR could report multiple ADRs, the proportion of ADRs relative to the total number of ICSRs was calculated. This proportion was used to compare the safety profiles of RDV and NMVr.

### 2.3. Disproportionality Analysis

In the EV database, ADRs are reported using preferred terms (PTs). A high-level term (HLT) can include multiple PTs. ADRs were categorized using the *Medical Dictionary for Regulatory Activities* (MedDRA), which classifies ADRs at different levels based on anatomy, pathology, physiology, etiology, or function. At the highest level, ADRs were grouped into 27 SOCs, each containing several high-level group terms (HLGTs) [22,23].

A disproportionality analysis was performed to compare RDV and NMVr at the SOC level. Additionally, comparisons at the HLT level were conducted for RDV and NMVr compared with other reference antivirals, including darunavir, sotrovimab, ritonavir, tenofovir, sofosbuvir, and ribavirin. The selection of comparator drugs in this study was based on their established clinical relevance, mechanisms of action (Table 1), and known safety profiles, particularly concerning neuropsychiatric ADRs. These drugs were chosen to provide a broader context for evaluating the neuropsychiatric effects observed with RDV and NMVr. Each of these antivirals has been previously approved for the treatment of various viral infections, with well-documented safety data, including reports of neuropsychiatric ADRs.

ADRs within the SOCs ‘Nervous system disorders’ and ‘Psychiatric disorders’ were particularly highlighted.

Specific ADRs targeted within these SOCs included:

- Psychiatric disorders: anxiety, delirium, depressed mood disorders, thinking and perception disorders, personality and behavioral disturbances, sleep disorders, and suicidal or self-harming behavior.
- Neurological disorders: headache, mental impairment, movement disorders, seizures, and sleep disorders.

A total of 46 PTs were analyzed for psychiatric disorders and 15 for neurological disorders (Table 2).

To determine disproportionality, ROR and 95% CI [24] were calculated using the MedCalc Software Ltd. The Odds Ratio Calculator (version 23.1.3) was used for this analysis [25]. Microsoft^®^ Excel^®^ for Microsoft 365 MSO (Version 2501)—Data Analysis Tools was used to performed descriptive analysis.

$$ROR=\frac{a\times d}{b\times c}$$

where

*ROR* = reporting odds ratio;

*a* = evaluated ADR for the targeted drug;

*b* = other ADRs for the targeted drug;

*c* = evaluated ADR for the drug used for comparison;

*d* = other ADRs for the drug used for comparison.

95% CI = exp (ln (*ROR*) − 1.96 × SE{ln(*ROR*)}) to exp (ln(*ROR*) + 1.96 × SE{ln(*ROR*)})

where

CI = confidence interval;

SE = standard error.

$$SE\{ln\left(ROR\right)\}=\sqrt{\frac{1}{a}+\frac{1}{b}+\frac{1}{c}+\frac{1}{d}}$$

The analysis compared RDV and NMVr with other antiviral drugs, following the European Medicines Agency (EMA) guidelines. According to EMA guidelines, a signal is defined as disproportionate when a minimum of five cases have been reported, and the lower bound of the 95% CI is greater than 1 [26].

### 2.4. Ethics

This study did not require ethical approval, as all reports in the EV portal are anonymized and do not include personal data.

## 3. Results

### 3.1. Descriptive Analysis of ICSRs

According to the data presented in Table 3, the highest frequency of reports was observed in the 18–64-year age group, with 37.7% of RDV and 38.0% of NMVr reports, followed by the 65–85 age group, with 35.5% and 38.5%, respectively. These age distributions reflect the predominant use of these drugs in adult and elderly populations, who are at higher risk of severe COVID-19 outcomes.

The proportions of ICSRs by sex showed an inverse distribution between the two drugs. In the female group, ICSRs for NMVr accounted for 61.8%, while those for RDV constituted only 36.8%. Conversely, male patients represented the majority of RDV reports, with 58.7%, compared to 33.9%, for NMVr. This reversed pattern may indicate differences in the clinical indications of the drugs, patterns of use, or possible gender differences in ADR reporting.

In terms of geographical distribution, most ICSRs were reported from non-EEA regions, with 64.3% of RDV reports and 51.6% of NMVr reports. This may reflect regional differences in drug availability, use policies, or reporting practices.

A significant difference was noted in the type of reporters. While HPs submitted the majority of ICSRs for both drugs, the proportion was significantly higher for RDV (93.4%) compared to NMVr (55.0%). This disparity suggests that NMVr, being an oral antiviral widely prescribed in outpatient settings, may have greater involvement from non-HPs, potentially affecting the depth and accuracy of ADR reporting.

There was also a notable difference in the severity of reported cases. Serious ICSRs accounted for 84.2% of the total reports for RDV compared to 59.9% for NMVr. This finding highlights the potentially more serious safety profile of RDV, probably related to its intravenous administration and systemic toxicities, in contrast to NMVr, which is predominantly used in less severe outpatient cases.

**Figure 1:**
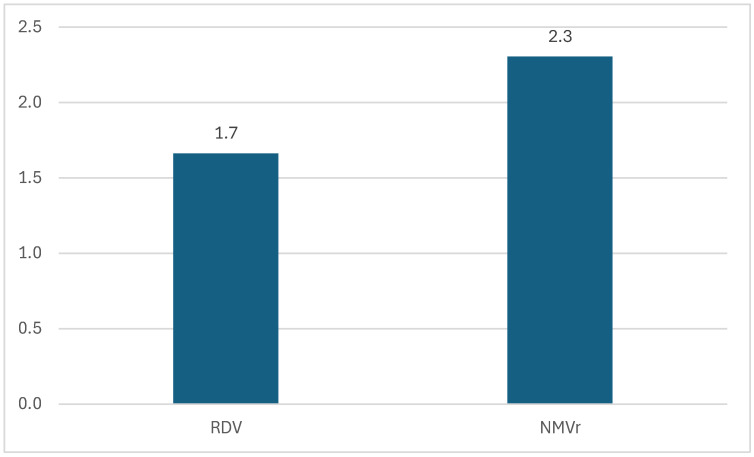
The proportion of ADRs reported from the total ICSRs. NMVr—nirmatrelvir/ritonavir; RDV—remdesivir.

For NMVr, a total of 8078 ICSRs were reported in the EV database, a value which is significantly higher compared to the 3934 ICSRs for RDV. Figure 1 shows a higher proportion of ADRs reported in the EV database for each case for NMVr (2.3) compared to RDV (1.7).

**Figure 2:**
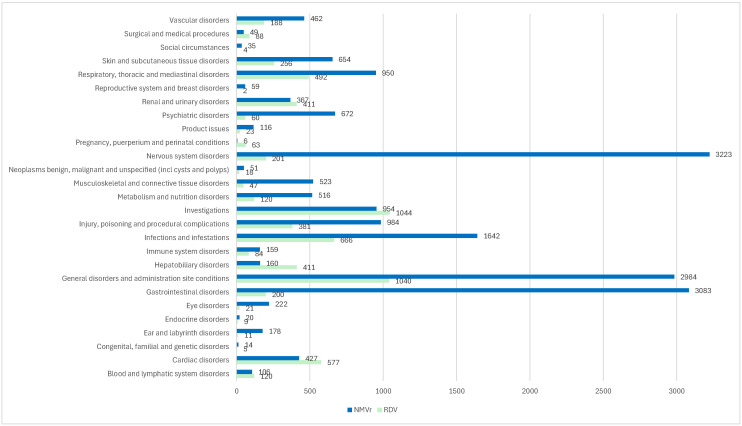
ADRs structured by SOC. NMVr—nirmatrelvir/ritonavir; RDV—remdesivir.

According to the data in Figure 2, most ADRs structured by SOC were reported for NMVr, with the exception of certain categories such as “Blood and lymphatic system disorders” (RDV: *n* = 120; NMVr: *n* = 106), “Cardiac disorders” (RDV: *n* = 577; NMVr: *n* = 427), “Hepatobiliary disorders” (RDV: *n* = 411; NMVr: *n* = 160), “Investigations” (RDV: *n* = 1044; NMVr: *n* = 954), “Pregnancy, puerperium and perinatal conditions” (RDV: *n* = 63; NMVr: *n* = 6), “Renal and urinary disorders” (RDV: *n* = 411; NMVr: *n* = 367) and “Surgical and medical procedures” (RDV: *n* = 88; NMVr: *n* = 49). These results suggest a higher incidence of systemic and procedural ADRs for RDV.

In contrast, NMVr had the highest number of ADRs in SOCs such as “Nervous system disorders” (*n* = 3223), “Gastrointestinal disorders” (*n* = 3083) and “General disorders and administration site conditions” (*n* = 2984). A significant difference in the proportion of ADRs compared to the total number of reports was observed for specific SOCs. For “Nervous system disorders”, RDV accounted for 201 cases (3.07%) compared to 3223 cases (17.31%) for NMVr. Similarly, for “Psychiatric disorders”, RDV contributed 60 cases (0.92%) compared to 672 cases (3.61%) for NMVr. These findings underscore NMVr’s more significant impact on the neuropsychiatric and gastrointestinal systems, whereas RDV’s ADRs are more concentrated in systemic categories such as hepatobiliary and renal disorders.

**Figure 3:**
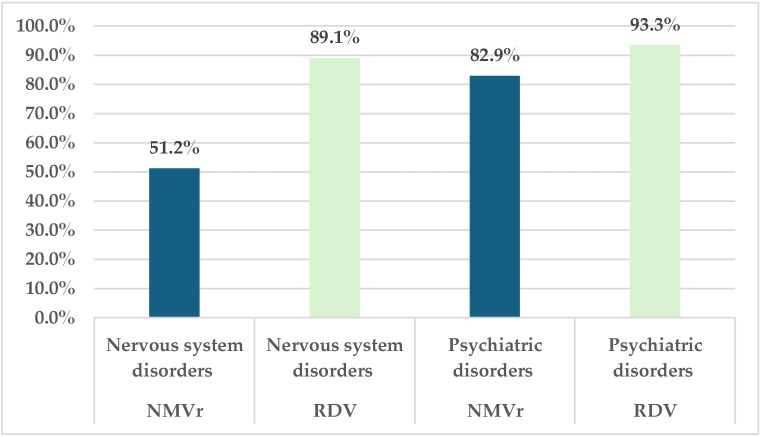
Frequency of the serious neuropsychiatric ADRs reported for RDV and NMVr. NMVr—nirmatrelvir/ritonavir; RDV—remdesivir.

Compared to RDV (93.3%), serious ADRs reported for NMVr represent 92.9% of the total psychiatric disorders. Regarding nervous system disorders, the difference between the two drugs is more pronounced (NMVr: 51.2% vs. RDV: 89.1%) (Figure 3).

### 3.2. Disproportionality Analysis

#### 3.2.1. Disproportionality Analysis of ADRs Grouped by SOCs

A comparison of the two antiviral drugs used in the COVID-19 treatment revealed notable differences in the incidence of ADRs in different SOCs. For RDV, a higher probability of ADRs was observed in the following SOCs: “Blood and lymphatic system disorders” (ROR: 3.263; 95% CI: 2.5089–4.2437), “Cardiac disorders” (ROR: 4.1205; 95% CI: 3.6236–4.6854), “Hepatobiliary disorders” (ROR: 7.7326; 95% CI: 6. 4272–9.3032), “Renal and urinary tract disorders” (ROR: 3.3334; 95% CI: 2.8872–3.8485), and “Respiratory, thoracic and mediastinal disorders” (ROR: 1.5123; 95% CI: 1.3511–1.6927). These results highlight the stronger association of RDV with systemic toxicities affecting vital organ systems.

In comparison, RDV showed a lower probability of ADRs compared to NMVr in specific SOCs, including “Ear and labyrinth disorders” (ROR: 0.1745; 95% CI: 0.0948–0.3210), “Eye disorders” (ROR: 0.2668; 95% CI: 0.1704–0.4178), “Gastrointestinal disorders” (ROR: 0. 1589; 95% CI: 0.1373–0.1839), “Metabolic and nutritional disorders” (ROR: 0.6555; 95% CI: 0.5363–0.8011), “Nervous system disorders” (ROR: 0.1514; 95% CI: 0.1309–0.1751), and “Psychiatric disorders” (ROR: 0.2472; 95% CI: 0.1895–0.3224) (Table 4). These results suggest that NMVr is more strongly associated with ADRs affecting the CNS, metabolism, and sensory organs.

#### 3.2.2. Disproportionality Analysis of ADRs Related to Psychiatric Disorders and Nervous System Disorders SOCs

**Figure 4:**
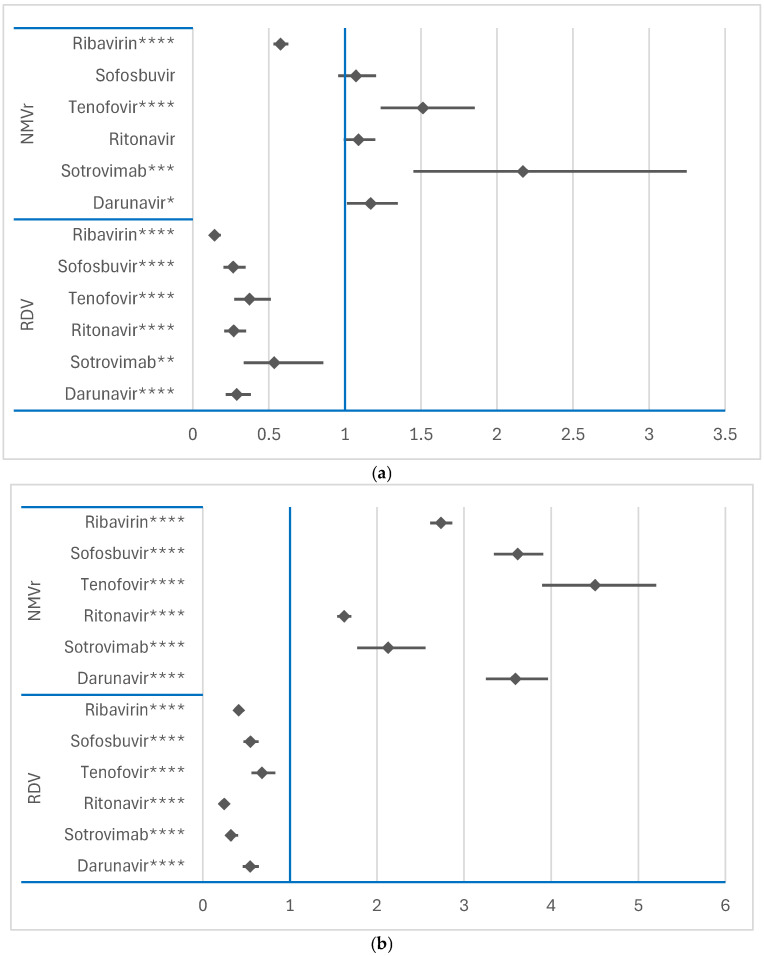
Disproportionality analysis of ADRs reported for RDV and NMVr in “Psychiatric disorders” and “Nervous system disorders” SOCs: (**a**)—“Psychiatric disorders” SOC; (**b**)—“Nervous system disorders” SOC; NMVr—nirmatrelvir/ritonavir; RDV—remdesivir. * *p* ≤ 0.05; ** *p* ≤ 0.01; *** *p* ≤ 0.001; **** *p* ≤ 0.0001.

For NMVr, psychiatric disorders were reported at a significantly higher frequency than for darunavir (ROR: 1.1690; 95% CI: 1.0137–1.3479), sotrovimab (ROR: 2.1706; 95% CI: 1.4509–3.2472) and tenofovir (ROR: 1.5127; 95% CI: 1.2352–1.8526), as shown in Figure 4a. Similarly, for the SOC “Nervous system disorders”, NMVr had a higher probability of ADR reporting than all other comparator drugs, as shown in Figure 4b. These findings highlight the distinct safety profile of NMVr, particularly its association with CNS-related ADRs.

However, RDV had a lower probability of ADR reporting in both the psychiatric and nervous system SOCs compared to the same reference drugs. These observations, presented in Figure 4a,b, further highlight the differences in the neuropsychiatric impact between NMVr and RDV, with NMVr showing a broader association with CNS ADRs.

#### 3.2.3. Disproportionality Analysis of Psychiatric ADRs

##### Comparison Between RDV or NMVr and Other Antivirals

**Figure 5:**
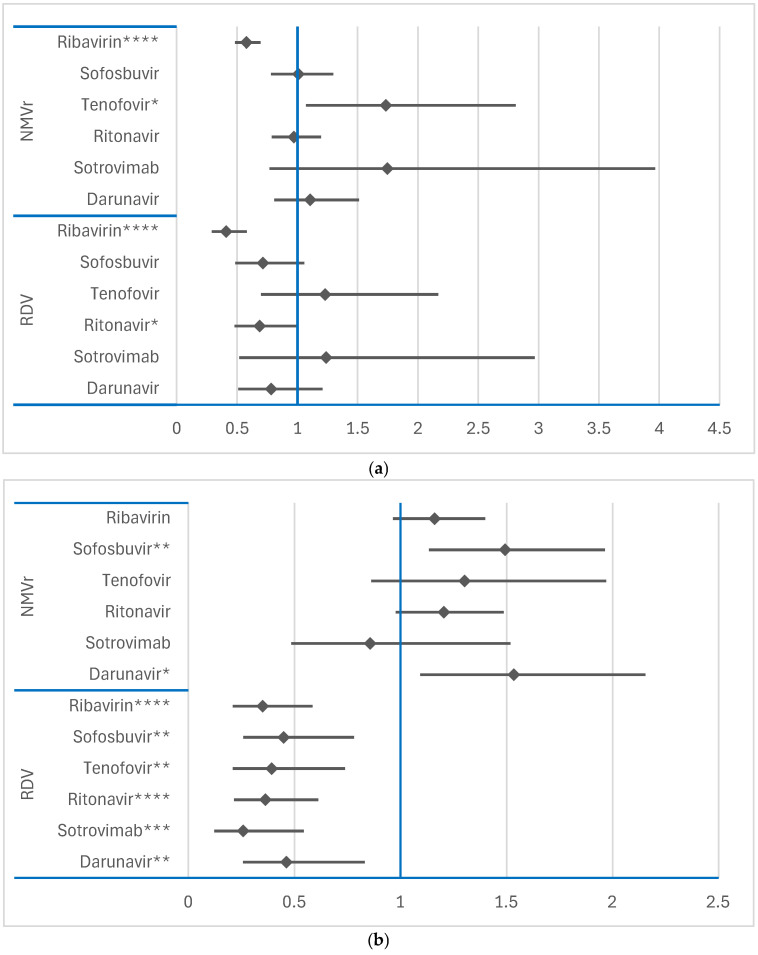

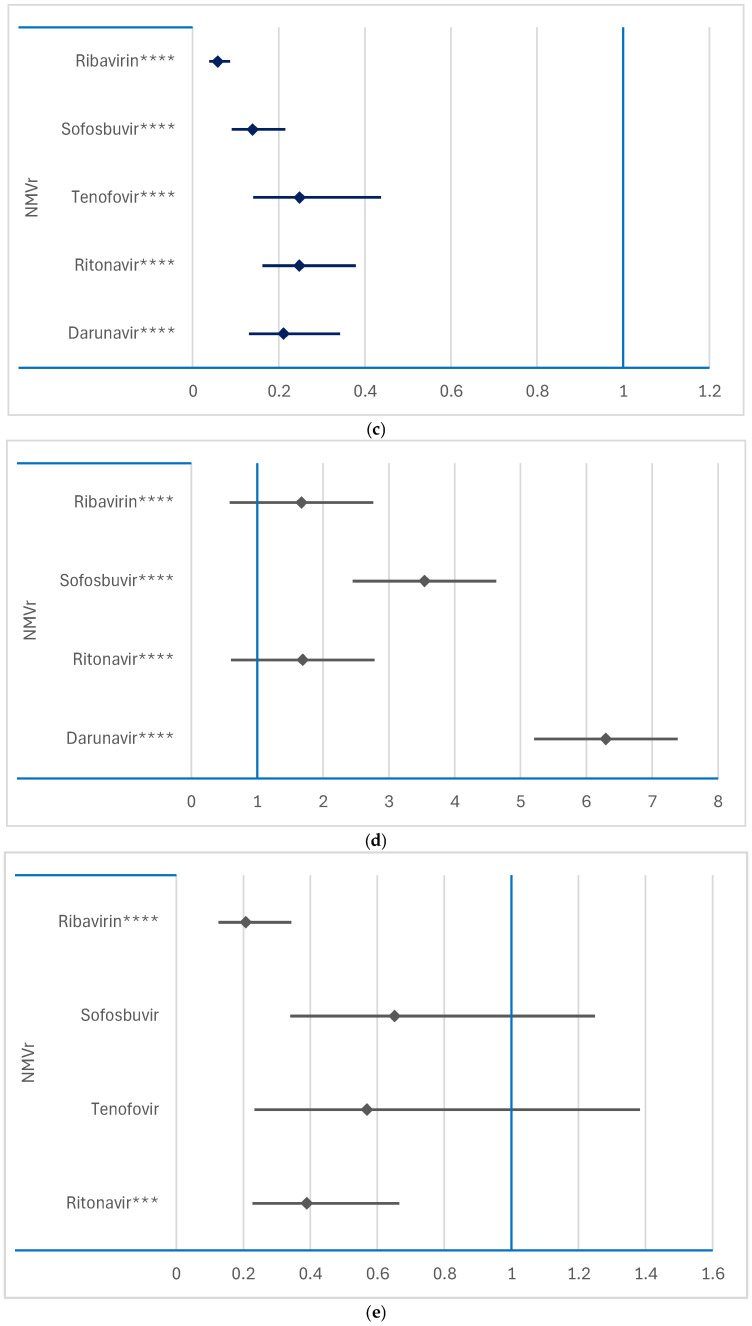

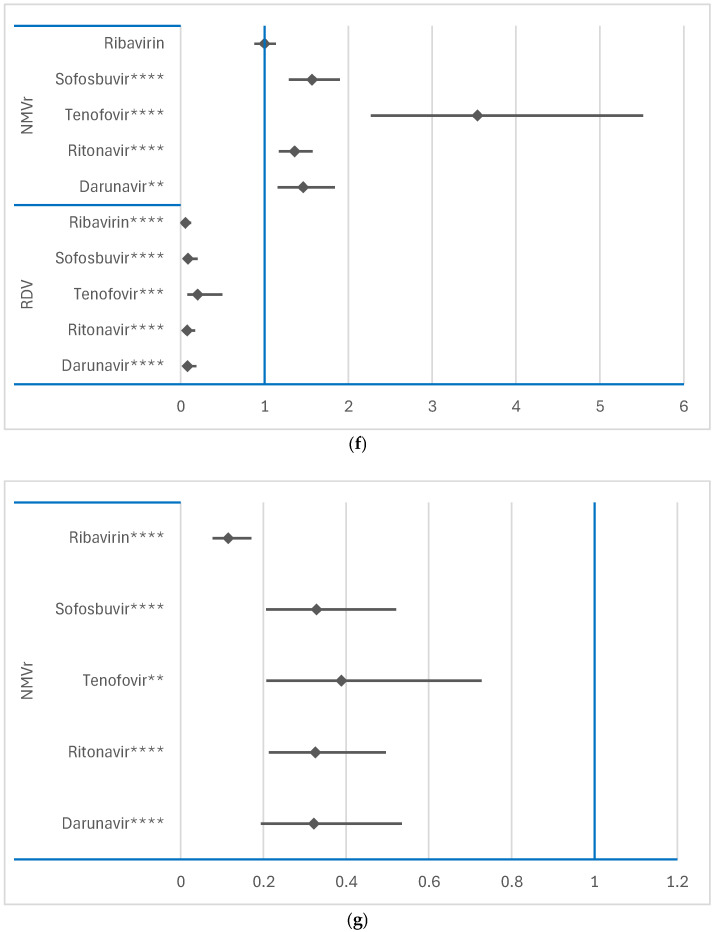
Disproportionality analysis for psychiatric disorders of remdesivir and ritonavir-boosted nirmatrelvir: (**a**) anxiety disorders and symptoms; (**b**) deliria (including confusion); (**c**) depressed mood disorders and disturbances; (**d**) disturbances in thinking and perception; (**e**) personality disorders and disturbances in behavior; (**f**) sleep disorders and disturbances; (**g**) suicidal and self-injurious behaviors NEC. NEC—not elsewhere classifiable; NMVr—nirmatrelvir/ritonavir; RDV—remdesivir. * *p* ≤ 0.05; ** *p* ≤ 0.01; *** *p* ≤ 0.001; **** *p* ≤ 0.0001.

NMVr has a higher probability of being associated with anxiety compared to tenofovir (ROR: 1.7353; 95% CI: 1.0716–2.8101), as shown in Figure 5a. Similarly, the reporting probability of delirium associated with NMVr is higher than that associated with darunavir (ROR: 1.5345; 95% CI: 1.0929–2.1547) and sofosbuvir (ROR: 1.4929; 95% CI: 1.1350–1.9637), as shown in Figure 5b. Cognitive disturbances were also more likely to be reported with NMVr compared to darunavir (ROR: 6.2932; 95% CI: 3.407–11.6244) and sofosbuvir (ROR: 3.5394; 95% CI: 2.4448–5.1241), as shown in Figure 5d. Sleep disorders were reported more frequently for NMVr compared to ritonavir (ROR: 1.3588; 95% CI: 1.1713–1.5763), tenofovir (ROR: 3.5385; 95% CI: 2.2702–5.5155), sofosbuvir (ROR: 1.5668; 95% CI: 1.292–1.9001), and darunavir (ROR: 1.4598; 95% CI: 1.1589–1.8388), as shown in Figure 5f.

In contrast, RDV does not show a higher reporting probability of adverse ADRs included in the HLTs of the psychiatric disorders analyzed, as shown in Figure 5a,b,f. RDV showed a lower reporting probability of delirium compared to all other drugs (Figure 5b) and sleep disorders (Figure 5f), except for sotrovimab. Similarly, anxiety (Figure 5a) was less likely to be reported for RDV compared to ritonavir and ribavirin. For NMVr, anxiety disorders were reported at a lower frequency compared to ribavirin. In addition, NMVr had a lower incidence of reporting depressive mood disorders (Figure 5c) and suicidal or self-harming behaviors (Figure 5g) compared to other drugs, with the exception of sotrovimab. Compared to the other drugs included in the analysis, NMVr was not associated with an increased risk of reporting ADRs related to personality disorders and behavioral disorders (Figure 5e).

##### Comparison Between RDV and NMVr

**Figure 6:**
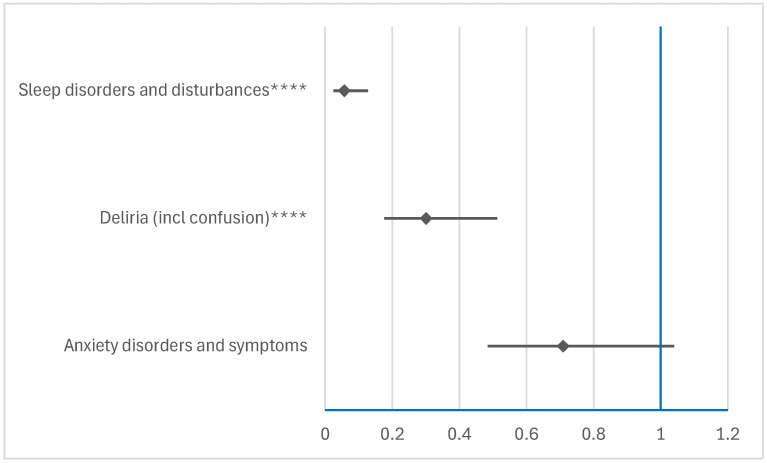
Disproportionality analysis of psychiatric ADRs reported for RDV compared to NMVr. **** *p* ≤ 0.0001.

From the analysis of all HLTs, a disproportionate signal was observed between RDV and NMVr for delirium (ROR: 0.3011; 95% CI: 0.1767–0.5132) and sleep disorders (ROR: 0.057; 95% CI: 0.0254–0.128). These results indicate a significantly lower reporting probability of ADRs for RDV compared to NMVr, as illustrated in Figure 6. This finding highlights the different safety profiles of the two drugs, particularly in relation to the incidence of neuropsychiatric ADRs.

### 3.3. Disproportionality Analysis of Neurological ADRs

#### 3.3.1. Comparison Between RDV or NMVr and Other Antivirals

**Figure 7:**
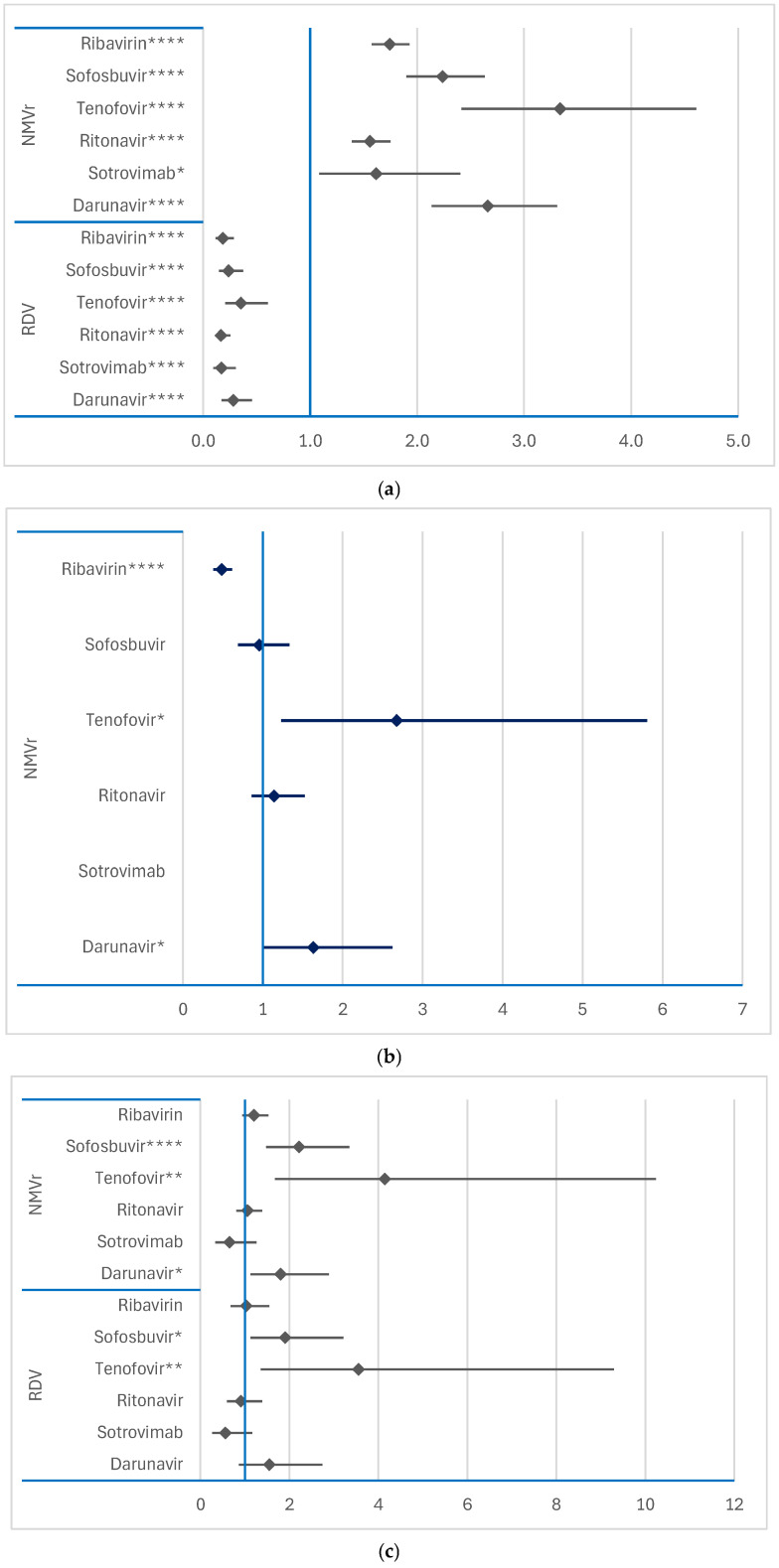

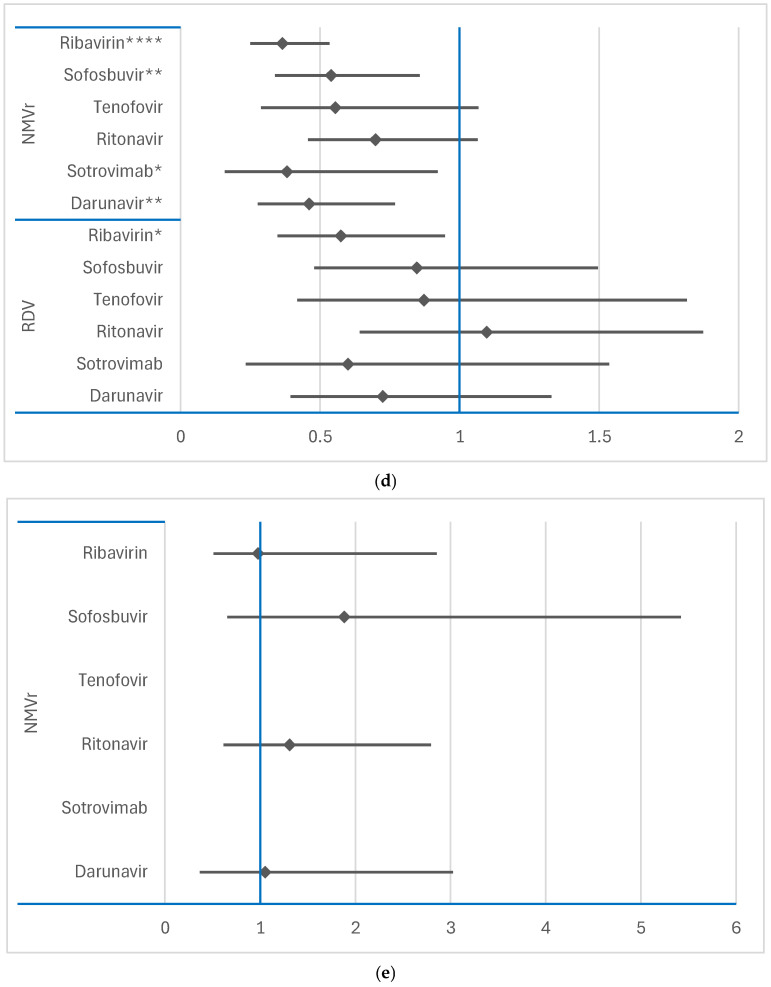
Disproportionality analysis for psychiatric disorders of remdesivir and nirmatrelvir/ritonavir: (**a**) headaches; (**b**) mental impairment disorders; (**c**) movement disorders; (**d**) seizures; (**e**) sleep disturbances. NMVr—nirmatrelvir/ritonavir; RDV—remdesivir. * *p* ≤ 0.05; ** *p* ≤ 0.01; **** *p* ≤ 0.0001.

NMVr was more likely to be associated with headache than any of the other drugs analyzed. However, RDV had a lower reporting probability of headache than any of the comparator drugs, as shown in Figure 7a. Regarding ADRs related to mental disorders, NMVr showed a higher probability compared to tenofovir only (ROR: 2.675; 95% CI: 1.232–5.8079), as shown in Figure 7b.

Many PTs (bradykinesia, dyskinesia, paralysis, psychomotor hyperactivity, tremor) have been used for reporting movement disorders. A higher reporting probability of movement disorders for both RDV and NMVr compared to specific antivirals was registered. For RDV, the reporting probability of movement disorders was higher compared to that of tenofovir (ROR: 3.553; 95% CI: 1.3591–9.2881) and sofosbuvir (ROR: 1.9054; 95% CI: 1.1283–3.2176). Similarly, NMVr was associated with a higher risk of reporting movement disorders compared to tenofovir (ROR: 4.148; 95% CI: 1.6812–10.2344), sofosbuvir (ROR: 2.2244; 95% CI: 1.4786–3.3464), and darunavir (ROR: 1.8054; 95% CI: 1.1276–2.8907) (Figure 7c).

Neither RDV nor NMVr showed a higher likelihood of reporting seizures (Figure 7d) or sleep disturbances (Figure 7e) compared to the other drugs analyzed. These findings highlight the unique neuropsychiatric safety profiles of RDV and NMVr, particularly their contrasting associations with headache and movement disorders.

#### 3.3.2. Comparison Between RDV and NMVr

**Figure 8:**
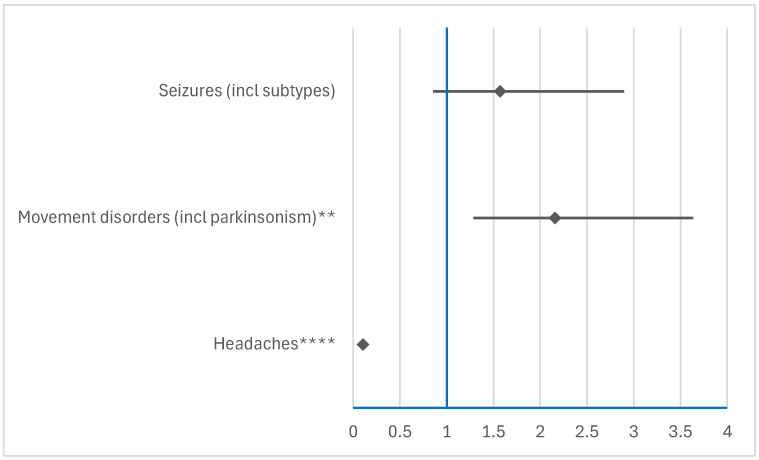
Disproportionality analysis of neurologic ADRs reported for RDV compared to NMVr. ** *p* ≤ 0.01; **** *p* ≤ 0.0001.

In the neurological category, RDV showed a higher frequency of reporting movement disorders compared to NMVr (ROR: 2.1602; 95% CI: 1.2837–3.6352). Furthermore, RDV showed a significantly lower frequency of reporting headache compared to NMVr (ROR: 0.1057; 95% CI: 0.0676–0.1653). These findings highlight the different neurological ADR profiles of the two antivirals, with RDV more likely to be associated with movement disorders and NMVr more likely to be associated with headaches (Figure 8).

## 4. Discussion

This study provides a comparative analysis of neuropsychiatric ADRs associated with RDV and NMVr-based ICSRs from the EV database. The results provide important insights into the safety profiles of these antivirals during their widespread use in the COVID-19 pandemic. By including other antivirals such as tenofovir, sofosbuvir, and darunavir as comparators, the study provides context for the observed ADRs and highlights the clinical implications of these therapies.

RDV and NMVr were selected for this analysis because of their central role in the treatment of COVID-19. RDV has been widely used to reduce the length of hospitalization and improve outcomes in severe COVID-19 cases [27,28], while NMVr has gained prominence for outpatient management due to its oral administration and efficacy against SARS-CoV-2 variants [29,30].

The comparator drugs were selected based on their mechanisms of action, therapeutic relevance, and known safety profiles, particularly concerning neuropsychiatric ADRs. Tenofovir, a nucleotide reverse transcriptase inhibitor, was selected because of its proven effectiveness in treating viral infections such as HIV and hepatitis B, where it has shown a relatively mild neuropsychiatric ADR profile. Its mechanism of action, targeting viral reverse transcriptase, minimizes its interaction with CNS pathways, making it a suitable reference for assessing the ADRs of more complex antiviral agents [31,32]. Similarly, sofosbuvir, an RNA polymerase inhibitor, is widely used in the treatment of hepatitis C and is recognized for its efficacy and limited neuropsychiatric ADRs [33,34,35]. By including sofosbuvir, the analysis benefited from comparison with an agent that has a similar molecular target to RDV but a different clinical use and safety profile.

Our findings confirm previous studies suggesting that tenofovir, a nucleotide reverse transcriptase inhibitor, has a relatively mild neuropsychiatric ADR profile compared to protease inhibitors. Also, the lower incidence of reported ADRs, such as delirium, associated with RDV compared to sofosbuvir (ROR: 0.4496; 95% CI: 0.2589–0.7805) underscores its distinct safety profile with respect to CNS-related effects. Moreover, NMVr was associated with a significantly higher reporting probability of sleep disorders compared to tenofovir (ROR: 3.5385; 95% CI: 2.2702–5.5155) and sofosbuvir (ROR: 1.5668; 95% CI: 1.292–1.9001).

Darunavir, a protease inhibitor, was included in the analysis to assess potential class-specific effects, particularly regarding its interaction with CNS pathways. Protease inhibitors, especially when combined with ritonavir for pharmacokinetic boosting, are known to affect cytochrome P450 enzymes, leading to significant drug–drug interactions and potential alterations to CNS homeostasis [36,37,38]. This selection allowed a focused evaluation of whether the ADRs observed in association with NMVr were consistent with the effects of the protease inhibitor class or were unique to this specific combination. By including these comparators, the study aimed to contextualize the neuropsychiatric ADRs observed with RDV and NMVr, providing a broader understanding of how these treatments compare to other antiviral therapies. This comparative approach also helps to determine whether the ADR profiles are driven by drug-specific characteristics or class-wide mechanisms, providing insights for safer clinical use.

A major finding was the disproportionately higher frequency of ADRs associated with NMVr in the “Nervous system disorders” and “Psychiatric disorders” SOCs. NMVr accounted for 17.31% and 3.61% of the total ADRs in these categories, respectively, compared to 3.07% and 0.92%, respectively, for RDV. According to our results, serious neuropsychiatric ADRs were reported less frequently for NMVr than for RDV. Specific psychiatric ADRs, including delirium (ROR: 0.3011; 95% CI: 0.1757–0.5132) and sleep disorders and disturbances (ROR: 0.057; 95% CI: 0.0254–0.128), were reported significantly more often in association with RDV than with NMVr. These results are in line with previous studies on protease inhibitors, highlighting their potential to induce neuropsychiatric effects via CYP3A4 inhibition, leading to drug–drug interactions and altered CNS homeostasis [16,17]. Patients receiving NMVr should undergo neuropsychiatric monitoring, particularly those with preexisting psychiatric conditions, as ritonavir can disrupt neurotransmitter metabolism via CYP3A4 inhibition, exacerbating anxiety and sleep disturbances [39]. Given the increased neuropsychiatric ADR risk, baseline psychiatric assessments and regular follow-ups are recommended, especially in polypharmacy cases. If severe ADRs arise, dose adjustments should be considered. Patient education on CNS effects and early symptom recognition is crucial, ensuring multidisciplinary collaboration for optimal management. RDV had a higher incidence of ADRs associated with systemic toxicity, particularly in the SOCs “Hepatobiliary disorders” (ROR: 7.7326; 95% CI: 6.4272–9.3032) and “Renal and urinary disorders” (ROR: 3.3334; 95% CI: 2.8872–3.8485). These findings are consistent with its known pharmacodynamic profile, where RDV metabolites concentrate in the kidneys and liver, potentially leading to organ-specific toxicities [40,41,42]. These ADRs highlight the importance of monitoring hepatic and renal function in patients receiving RDV, particularly those with preexisting comorbidities [43].

Furthermore, the inclusion of ribavirin and sotrovimab as comparator drugs was to increase the depth of the ADR profile comparison, given their different pharmacological classes and ADR profiles.

Ribavirin, a nucleoside analog used for hepatitis C and viral hemorrhagic fevers, has also been associated with neuropsychiatric effects. Common ADRs include fatigue, irritability, and depression. In rare cases, ribavirin use has been linked with severe psychiatric conditions, including suicidal ideation and psychosis, highlighting the importance of careful patient monitoring [44,45]. Our study found that NMVr has a significantly higher reporting probability of neuropsychiatric ADRs compared to ribavirin, particularly in categories such as disturbances in thinking and perception (ROR: 1.6708, 95% CI: 1.3794–2.0238), headaches (ROR: 1.7434; 95% CI: 1.5748–1.9302), and neurological disorders (ROR: 5.2754; 95% CI: 4.9857–5.582). Sotrovimab, a monoclonal antibody designed to neutralize SARS-CoV-2 by targeting a conserved epitope of the spike protein, has gained attention for its role in preventing severe outcomes in high-risk COVID-19 patients [46]. The ADRs associated with sotrovimab are predominantly mild and systemic, such as infusion-related reactions, fever, and fatigue. However, there is emerging evidence of occasional neuropsychiatric manifestations, including headache, anxiety, and transient confusion. The drug’s mechanism of action, which focuses on viral neutralization rather than enzymatic inhibition, contributes to its generally favorable safety profile, but long-term data remain limited [47,48,49]. In our findings, sotrovimab provided a valuable contrast, particularly for neurologic ADRs such as seizures, where its low reporting odds further highlighted the CNS-related risks of NMVr. These findings highlight the importance of considering drug-specific safety profiles when managing COVID-19, particularly in patients with preexisting neurologic disorders.

Headache was reported significantly more often in association with NMVr than with any other antiviral (including RDV). This finding suggests a possible CNS mechanism influenced by the pharmacokinetic enhancement of ritonavir, which may amplify the effects of NMVr on serotonergic and dopaminergic pathways [50]. This hypothesis is consistent with existing literature on ritonavir-boosted regimens and their association with neuropsychiatric ADRs [51,52].

There were also gender differences in ADR reporting, with a higher proportion of female patients reporting ADRs for NMVr (61.8%) compared to RDV (36.8%). This could be explained by pharmacokinetic differences between the sexes, such as differences in cytochrome P450 enzyme activity and body composition, both of which affect drug metabolism and distribution [53]. Further studies are needed to investigate these sex-related pharmacodynamic differences in order to optimize dosing strategies for female patients.

It is also worth noting the differences in the severity of ADRs between the two drugs. Serious ADRs represented 84.2% of RDV-related ICSRs compared to 59.9% for NMVr. This difference may be partly explained by the intravenous administration of RDV and its use in more severe cases, where systemic ADRs are more likely to be documented by HPs. The higher proportion of non-serious ADRs for NMVr, an oral antiviral predominantly prescribed in outpatient settings, reflects its wider accessibility and use in less critically ill populations.

Our findings indicate that RDV has a relatively lower incidence of neuropsychiatric ADRs, aligning with previous studies that did not highlight significant CNS-related effects, instead emphasizing hepatic and renal concerns, likely due to its metabolic clearance and low BBB penetration [54]. In contrast, NMVr was associated with a higher likelihood of neuropsychiatric ADRs, such as anxiety and sleep disturbances, in alignment with pharmacokinetic data suggesting that ritonavir’s CYP3A4 inhibition may exacerbate CNS effects by increasing drug exposure and altering neurotransmitter metabolism [55]. Furthermore, reports from the FDA Adverse Event Reporting System (FAERS) and real-world cohort studies support our findings, linking NMVr to increased neuropsychiatric symptoms, particularly in patients with underlying psychiatric conditions or polypharmacy risks [39,56]. These results emphasize the need for careful monitoring of NMVr, particularly in vulnerable populations, to mitigate potential CNS-related ADRs.

The neuropsychiatric adverse ADRs observed in this study may be explained by several mechanisms, including direct CNS penetration, drug interactions, and neuroinflammation. SARS-CoV-2 itself exhibits neuroinvasive potential, with mechanisms such as cytokine-induced neuroinflammation, endothelial damage, and direct viral entry into neural tissue contributing to a wide spectrum of neuropsychiatric manifestations. These include mood disturbances, cognitive impairments, and persistent fatigue, all of which have been reported in COVID-19 patients independent of the antiviral treatment. However, the pharmacological properties of RDV and NMVr suggest additional pathways through which these drugs might influence CNS function [5].

One possible mechanism involves direct CNS penetration. While RDV and NMVr were not primarily designed to target the CNS, their pharmacokinetic profiles suggest that they may cross the BBB, particularly in the context of SARS-CoV-2 infection. COVID-19-related inflammation and endothelial damage can increase BBB permeability, allowing a higher concentration of drugs to enter the CNS. This increased penetration may contribute to neuropsychiatric symptoms, particularly in individuals with preexisting vulnerabilities or those receiving prolonged antiviral therapy [17,57].

Another key mechanism is related to drug interactions. NMVr contains ritonavir, a strong inhibitor of cytochrome P450 enzymes, particularly CYP3A4, which plays a crucial role in drug metabolism. By inhibiting CYP3A4, ritonavir can elevate the plasma concentrations of concomitantly administered medications, increasing the likelihood of neuropsychiatric ADRs such as anxiety, delirium, and sleep disturbances. Previous studies on protease inhibitors have demonstrated their potential to induce CNS-related effects, including depression and cognitive impairment, likely due to altered neurotransmitter metabolism and interference with neuroendocrine signaling pathways [57].

Additionally, neuroinflammation remains a critical factor in the development of neuropsychiatric symptoms in COVID-19 patients undergoing antiviral treatments. SARS-CoV-2 infection induces a systemic inflammatory response, leading to the release of pro-inflammatory cytokines such as interleukin-6 and tumor necrosis factor-α. This cytokine storm has been linked to neuronal dysfunction and exacerbation of psychiatric disorders. The use of antiviral drugs in this inflammatory state may modulate neuroinflammatory pathways, potentially amplifying or mitigating CNS effects depending on the individual’s immune response [5,57,58].

### Limitations of the Study and Future Perspectives

A notable limitation of this study is the reliance on spontaneous ADR reports in the EV database, which may be subject to reporting bias and underreporting. EV is a spontaneous reporting system designed to collect adverse reactions resulting from real-world drug use through a self-reporting process. For this reason, the quality of the reports cannot be guaranteed. These reports may contain inaccurate data primarily due to the reporter, who can be healthcare professionals (physicians, pharmacists, etc.), or non-healthcare professionals (patients, caregivers). Moreover, the publicly accessible data available for research and public health protection do not provide information on the management of adverse reactions caused by drug administration. At the same time, underreporting and the unknown total population size using the drug make it difficult to calculate the incidence of adverse reactions.

We did not evaluate a causal relationship between the suspected drugs and the spontaneously reported adverse reactions, as these systems do not allow for such an assessment. However, our study establishes several important and useful statistical associations. Nonetheless, conducting disproportionality analysis from spontaneous reporting platforms is now a validated and widely used method in post-marketing drug safety surveillance. It is important to mention that this type of approach should be considered exploratory and should be supplemented with additional data obtained from interventional or observational studies or biological investigations.

## 5. Conclusions

Neuropsychiatric ADRs associated with antiviral treatments for COVID-19, particularly RDV and NMVr, are a critical area of investigation given their wide use during the pandemic. This analysis of the EV database highlights the different safety profiles of the two drugs. NMVr was associated with a significantly higher incidence of psychiatric ADRs, including delirium and sleep disorders, consistent with the known effects of protease inhibitors on CNS pathways. In most cases, RDV had a lower reporting probability of neuropsychiatric ADRs but was associated with a higher risk of systemic toxicities, particularly hepatobiliary and renal dysfunction. These findings highlight the importance of individualized patient monitoring during antiviral therapies. For NMVr, neuropsychiatric monitoring is essential, especially in patients with preexisting neurologic disorders (e.g., seizures) or those taking medications with potential CNS interactions. For RDV, liver and kidney function should be regularly monitored to reduce risk, especially in critically ill patients. A detailed patient history, including previous psychiatric or systemic comorbidities, drug interactions, and details of drug administration, is necessary for an effective management of ADRs. Moreover, clinical investigations, such as routine CNS assessments for NMVr and hepatic and renal monitoring for RDV, should be incorporated into treatment protocols. Dose adjustments or temporary treatment interruptions may be necessary to minimize risks and improve patient safety. This study highlights the role of pharmacovigilance databases in identifying real-world ADR patterns, informing clinical practice and guiding future research. Extending this work with pharmacogenomic studies and long-term follow-ups may provide deeper insights into ADR risks, enabling personalized antiviral therapies and improved patient outcomes.

## Figures and Tables

**Table 1:** Comparison of key findings, mechanisms of action, and pharmacological classes of the antiviral agents included in the study.

Antiviral Agent	Pharmacological Class	Mechanism of Action
**Remdesivir (RDV)**	RNA-dependent RNA polymerase inhibitor	Inhibits viral RNA polymerase, blocking viral replication.
**Nirmatrelvir/ritonavir (NMVr)**	Protease inhibitor + pharmacokinetic booster	Inhibits SARS-CoV-2 main protease; ritonavir boosts nirmatrelvir’s bioavailability.
**Ribavirin**	Nucleoside analog antiviral	Mimics natural nucleosides, inducing lethal mutagenesis in viral RNA.
**Sofosbuvir**	Nucleotide RNA polymerase inhibitor	Inhibits HCV RNA polymerase, leading to inhibition of viral replication.
**Tenofovir**	Nucleotide reverse transcriptase inhibitor	Blocks reverse transcriptase, preventing viral DNA synthesis in retroviruses.
**Darunavir**	HIV-1 protease inhibitor	Inhibits HIV protease, preventing the cleavage of polyproteins necessary for viral maturation.
**Sotrovimab**	Neutralizing monoclonal antibody	Targets the spike protein of SARS-CoV-2, preventing cell entry and viral replication.

**Table 2:** PTs used for reporting psychiatric and neurologic ADRs.

ADR Category	HLT	PT
**Psychiatric disorders**	Anxiety disorders and symptoms	Agitation
		Anxiety
		Fear
		Nervousness
		Panic attack
		Phobia of driving
		Stress
	Deliria (including confusion)	Confusional state
		Delirium
		Disorientation
	Depressed mood disorders and disturbances	Depressed mood
		Depression
		Depression suicidal
	Disturbances in thinking and perception	Autoscopy
		Bradyphrenia
		Delusion
		Hallucination
		Hallucination, auditory
		Hallucination, visual
		Tachyphrenia
		Thinking abnormal
	Mood disorders and disturbances NEC	Emotional distress
		Euphoric mood
		Frustration tolerance decreased
		Irritability
		Mood altered
		Mood swings
	Personality disorders and disturbances in behavior	Aggression
		Paranoia
	Sleep disorders and disturbances	Abnormal dreams
		Abnormal sleep-related event
		Hypnagogic hallucination
		Initial insomnia
		Insomnia
		Middle insomnia
		Nightmare
		Parasomnia
		Poor quality sleep
		Sleep disorder
		Sleep talking
		Sleep terror
		Sleep-related eating disorder
		Somnambulism
	Suicidal and self-injurious behaviors NEC	Completed suicide
		Suicidal ideation
		Suicide attempt
**Neurologic disorders**	Headaches	Headache
		Migraine
		Migraine with aura
	Mental impairment disorders	Amnesia
		Disturbance in attention
		Memory impairment
	Movement disorders	Bradykinesia
		Dyskinesia
		Freezing phenomenon
		Paralysis
		Psychomotor hyperactivity
		Resting tremor
		Tremor
	Seizures	Seizure
		Seizure like phenomena

**Table 3:** Characteristics of ICSRs reported for remdesivir and nirmatrelvir/ritonavir. EEA—European Economic Area; HP—healthcare professional; NMVr—ritonavir/nirmatrelvir; NS—not specified; RDV—remdesivir.

		RDV		NMVr	
		*n*	%	*n*	%
**Total**		**3934**	100.0%	**8078**	100.0%
Age category	NS	580	14.7%	1269	15.7%
	0–1 month	4	0.1%	5	0.1%
	2 months–2 years	22	0.6%	0	0.0%
	3–11 years	26	0.7%	0	0.0%
	12–17 years	28	0.7%	18	0.2%
	18–64 years	1483	37.7%	3072	38.0%
	65–85 years	1398	35.5%	3110	38.5%
	More than 85 years	393	10.0%	604	7.5%
Sex	Female	1448	36.8%	4996	61.8%
	Male	2310	58.7%	2735	33.9%
	NS	176	4.5%	347	4.3%
Geographic region	EEA	1406	35.7%	3912	48.4%
	NON-EEA	2528	64.3%	4166	51.6%
	NS	0	0.0%	0	0.0%
Reporter	HP	3673	93.4%	4445	55.0%
	Non-HP	261	6.6%	3633	45.0%
	NS	0	0.0%	0	0.0%
Seriousness	Non serious	621	15.8%	3239	40.1%
	NS	0	0.0%	0	0.0%
	Serious	3313	84.2%	4839	59.9%

**Table 4:** Disproportionality analysis of ADRs grouped by SOCs reported for RDV and NMVr. ADR—adverse drug reaction; CI—confidence interval; ROR—reporting odds ratio; NMVr—nirmatrelvir/ritonavir; RDV—remdesivir. * *p* ≤ 0.05; ** *p* ≤ 0.01; **** *p* ≤ 0.0001.

	ADRs of RDV	Other ADRs of RDV	ADRs of NMVr	Other ADRs of NMVr	ROR	95% CI Minimum	95% CI Maximum	*p*
Blood and lymphatic system disorders ****	120	6422	106	18 , 510	3.263	2.5089	4.2437	*p* < 0.0001
Cardiac disorders ****	577	5965	427	18 , 189	4.1205	3.6236	4.6854	*p* < 0.0001
Congenital, familial, and genetic disorders	5	6537	14	18,602	1.0163	0.3659	2.8227	*p* = 0.9752
Ear and labyrinth disorders ****	11	6531	178	18 , 438	0.1745	0.0948	0.3210	*p* < 0.0001
Endocrine disorders	9	6533	20	18,596	1.2809	0.5830	2.8145	*p* = 0.5376
Eye disorders ****	21	6521	222	18 , 394	0.2668	0.1704	0.4178	*p* < 0.0001
Gastrointestinal disorders ****	200	6342	3083	15 , 533	0.1589	0.1373	0.1839	*p* < 0.0001
General disorders and administration site conditions	1040	5502	2984	15,632	0.9902	0.9169	1.0694	*p* = 0.8022
Hepatobiliary disorders ****	411	6131	160	18 , 456	7.7326	6.4272	9.3032	*p* < 0.0001
Immune system disorders **	84	6458	159	18 , 457	1.5099	1.1574	1.9698	*p* = 0.0024
Infections and infestations **	666	5876	1642	16 , 974	1.1717	1.0657	1.2882	*p* = 0.0011
Injury, poisoning, and procedural complications	381	6161	984	17,632	1.1081	0.9811	1.2516	*p* = 0.0985
Investigations ****	1044	5498	954	17 , 662	3.5155	3.2038	3.8576	*p* < 0.0001
Metabolism and nutrition disorders ****	120	6422	516	18 , 100	0.6555	0.5363	0.8011	*p* < 0.0001
Musculoskeletal and connective tissue disorders ****	47	6495	523	18 , 093	0.2503	0.1855	0.3379	*p* < 0.0001
Neoplasms benign, malignant, and unspecified (including cysts and polyps)	18	6524	51	18,565	1.0043	0.5864	1.7202	*p* = 0.9874
Nervous system disorders ****	201	6341	3223	15 , 393	0.1514	0.1309	0.1751	*p* < 0.0001
Pregnancy, puerperium, and perinatal conditions ****	63	6479	6	18 , 610	30.1597	13.0479	69.7131	*p* < 0.0001
Product issues *	23	6519	116	18 , 500	0.5627	0.3594	0.8809	*p* = 0.0119
Psychiatric disorders ****	60	6482	672	17 , 944	0.2472	0.1895	0.3224	*p* < 0.0001
Renal and urinary disorders ****	411	6131	367	18 , 249	3.3334	2.8872	3.8485	*p* < 0.0001
Reproductive system and breast disorders	2	6540	59	18,557	NA	NA	NA	NA
Respiratory, thoracic, and mediastinal disorders ****	492	6050	950	17 , 666	1.5123	1.3511	1.6927	*p* < 0.0001
Skin and subcutaneous tissue disorders	256	6286	654	17,962	1.1185	0.9653	1.2961	*p* = 0.1362
Social circumstances	4	6538	35	18,581	NA	NA	NA	NA
Surgical and medical procedures ****	88	6454	49	18 , 567	5.1665	3.6389	7.3354	*p* < 0.0001
Vascular disorders	188	6354	462	18,154	1.1626	0.9790	1.3808	*p* = 0.0859

The blue color represents the lower reporting probability of ADRs for RDV; the red color represents the higher reporting probability of ADRs for NMVr.

## Data Availability

Data are contained within the article.

## Abbreviations

The following abbreviations are used in this manuscript:

ADR	Adverse drug reaction
CNS	Central nervous system
CI	Confidence interval
COVID-19	Coronavirus disease 2019
DNA	Deoxyribonucleic acid
EEA	European Economic Area
EMA	European Medicines Agency
EV	EudraVigilance
HCV	Hepatitis C virus
HLGT	High-level group term
HLT	High-level term
HP	Healthcare professional
HIV	Human immunodeficiency virus
ICSR	Individual case safety report
MedDRA	Medical Dictionary for Regulatory Activities
NEC	Not elsewhere classifiable
NMVr	Nirmatrelvir/ritonavir
NS	Not specified
RdRp	RNA-dependent RNA polymerase
RDV	Remdesivir
RNA	Ribonucleic acid
ROR	Reporting odds ratio
SARS-CoV-2	Severe acute respiratory syndrome coronavirus 2
SOC	System organ class
